# Mycobacterial MMAR_2193 catalyzes *O*-methylation of diverse polyketide cores

**DOI:** 10.1371/journal.pone.0262241

**Published:** 2022-01-05

**Authors:** Gorkha Raj Giri, Priti Saxena

**Affiliations:** Chemical Biology Group, Faculty of Life Sciences and Biotechnology, South Asian University, Akbar Bhawan, Chanakyapuri, New Delhi, India; Council for Scientific and Industrial Research, INDIA

## Abstract

*O*-methylation of small molecules is a common modification widely present in most organisms. Type III polyketides undergo *O*-methylation at hydroxyl end to play a wide spectrum of roles in bacteria, plants, algae, and fungi. *Mycobacterium marinum* harbours a distinctive genomic cluster with a type III *pks* gene and genes for several polyketide modifiers including a methyltransferase gene, *mmar_2193*. This study reports functional analyses of MMAR_2193 and reveals multi-methylating potential of the protein. Comparative sequence analyses revealed conservation of catalytically important motifs in MMAR_2193 protein. Homology-based structure-function and molecular docking studies suggested type III polyketide cores as possible substrates for MMAR_2193 catalysis. *In vitro* enzymatic characterization revealed the capability of MMAR_2193 protein to utilize diverse polyphenolic substrates to methylate several hydroxyl positions on a single substrate molecule. High-resolution mass spectrometric analyses identified multi-methylations of type III polyketides in cell-free reconstitution assays. Notably, our metabolomics analyses identified some of these methylated molecules in biofilms of wild type *Mycobacterium marinum*. This study characterizes a novel mycobacterial *O*-methyltransferase protein with multi-methylating enzymatic ability that could be exploited to generate a palette of structurally distinct bioactive molecules.

## Introduction

Methylation is an important biological activity with implications in several cellular processes including DNA repair, signal transduction, regulation of hormones and neurotransmitters, and secondary metabolites biosynthesis. Methyltransferases (MTases) in an enzymatic mechanism catalyze nucleophilic substitution reaction by the transfer of methyl group from universal donor S-Adenosyl *L*-methionine (SAM) to a nucleophile containing carbon (C), sulfur (S), nitrogen (N) and oxygen (O) [1]. Methylation in secondary metabolic pathways occurs on several biological molecules affecting the natural properties of the final products [2]. Based on the nucleophiles targeted for methylation, MTases are termed as C-methyltransferases (CMTs), S-methyltransferases (SMTs), N-methyltransferases (NMTs) and the most abundantly found, *O*-methyltransferases (OMTs). OMTs methylate the electron-rich *O*-position of the acceptor molecule and are present in diverse organisms, including plants, animals, bacteria, and fungi conferring a wide range of biological functions [3]. Two classes of OMTs are reported based on protein sequence and phylogenetic analysis [4]. Class I proteins with a size of 23–28 kDa require divalent cation Mg^2+^ as a cofactor [5] to organize the substrate-binding site [6]. It includes alfalfa (*Medicago sativa*) caffeoyl coenzyme A 3-OMT (CCoAOMT) [7], rat (*Rattus norvegicus*) catechol OMT (COMT) [8], different variants of repair enzyme protein of humans [9], *L*-isoaspartyl (*D*-aspartyl) OMTs from *Pyrococcus furiosus* [10] and *Vibrio cholerae* [11], and *Streptomyces clavuligerus* Cmcl [12]. Among the OMTs, catechol *O*-methyltransferases (COMTs) are most widely studied [8]. COMTs methylate hydroxyl moiety of catechol, neurotransmitters, and xenobiotics and have a role in inactivating the catechol-type compounds such as L-Dopamine [13]. Plant OMTs biosynthesize specialized metabolites like lignin from methylated caffeoyl-CoA, flavonoids, phenylpropanoid conjugate from methylation of phenolic compounds [14]. Class II MTases with larger subunit sizes (38-43kDa) involves a catalytic histidine residue in place of Mg^2+^ for methylation. This class includes alfalfa chalcone OMT [15] that carries out single methylation at 2’-position of isoliquiritigenin (4, 2’, 4’-trihydroxychalcone) giving 4, 4’-dihydroxy-2’-methoxychalcone, a signalling molecule and an inducer of nodulation in soil rhizobia [16, 17]. Class II OMTs also include isoflavone OMT [15] and caffeic acid OMT [18] from alfalfa, and isoflavonoid OMT from *Medicago truncatula* [19]. The domain analysis of Class II methyltransferases shows C-terminal end with a role in catalysis and N-terminal for dimerization of protein [20].

Class I AdoMet-dependent OMTs are also present in bacterial species like *Leptospira interrogans* (LiOMTs) [21] and *Bacillus cereus* (BcOMT2) [20]. Methyltransferase protein CheR1 along with flagella mediated chemotaxis is shown to be essential for biofilm generation and maintenance in *Pseudomonas aeruginosa* [22]. Methyltransferase proteins encoded by *Rv2952* and *Rv2959c* are also implicated in methylation of pathogenicity determinants like phenolglycolipids (PGL), dimycocerosate of phthiocerol (DIM) and related *p*-hydroxybenzoic acid derivatives (*p-*HBAD). These molecules upon methylation increase the virulence of pathogenic *M*. *tuberculosis* [23].

Bacteria produce an array of biologically potent aromatic and polyphenolic products from important class of enzymes called type III polyketide synthases (PKSs). These proteins catalyze condensation of an acyl-CoA starter substrate, iteratively with malonyl-CoA and/or methylmalonyl-CoA extender units. The type III polyketide products get variously modified for biological roles. In a recent study, *M*. *smegmatis* is shown to harbour an alkyl benzoquinone biosynthetic cluster comprising of a type III PKS (MSMEG_0808), methyltransferase (MSMEG_0809) and oxidoreductase (MSMEG_0809) [24]. Proteins from the cluster biosynthesize methylated polyketide quinones (PKQs) that aid anaerobic respiration in mycobacterial biofilms.

In this study, we have biochemically characterized a methyltransferase, *mmar_*2193, from a type III *pks* gene cluster in *Mycobacterium marinum* (Mmar). MMAR_2193 protein works as an *O*-methyltransferase, and high-resolution mass spectrometry revealed its capability to methylate hydroxyl positions in polyketide core molecules. MMAR_2193 could methylate multiple hydroxyl positions on a single substrate molecule to produce multimethylated products. Our metabolomics analyses identified some of these methylated products in wild type *Mycobacterium marinum* biofilms. This study thus characterizes a novel methyltransferase from Mmar with multi-methylation potential that could be utilized to generate unusual biologically active compounds.

## Materials and methods

### Bacterial strains and materials

We used *Escherichia coli* XL-1 blue and BL21 (DE3) as bacterial hosts for cloning and expression, respectively. Wild-type *M*. *marinum* (strain ATCC BAA-535/M) kindly provided by Prof. Y. Singh (IGIB, India) was grown on Middlebrook 7H9, Middlebrook 7H11 media. The strain was further used for genomic DNA isolation and for biofilm growth on Sauton’s Fluid Media Base. The MtbPKS18 expression clone was kindly gifted by R. S. Gokhale (NII, India) and was used for substrate biosynthesis. S-adenosyl *L*-methionine (SAM), acyl-CoA starter and extender substrates were procured from Sigma. *Escherichia coli* strains were grown on LB medium. Restriction endonucleases and PCR master mixture procured from New England Biolabs. PCR cleanup kit and Ni^2+^-NTA agarose were purchased from Qiagen. UPLC and MS grade solvents were purchased from Merck and Sigma.

### Genomic DNA isolation

Genomic DNA was isolated from *M*.*marinum* culture. The bacterial culture was streaked onto 7H11 Agar Medium with ADC supplement. Genomic DNA was isolated using the boiling method. Colonies of bacterial cells were scrapped and resuspended in 100μl water in microcentrifuge (MCT) tube. To the mixture equal volume of chloroform was added, vortexed and boiled for 10-15mins. Chloroform was again added, and the mixture was heated to evaporate the solvent followed by the addition of 100μl autoclaved water. The mixture was centrifuged to pellet down cell debris, proteins and lipids, and the supernatant containing genomic DNA was collected.

### Cloning of gene and sequence analysis

Bacterial genomic DNA was used as a template to amplify methyltransferase gene using a set of gene specific forward primer (5’**TTCAT**ATGGATTTTGATGCG CTG3’) containing NdeI restriction enzyme site and reverse primer (5’**TTAAGCTT**GTTTTGCCGCCGCGC3’) containing HindIII restriction site. The *mmar_2193* gene was amplified and cloned into pBLuescriptSK(+) cloning vector (Stratagene). Identity of the clone was confirmed by restriction digestion and automated nucleotide sequencing. The *mmar_2193* gene was further sub-cloned into pET 21c (Novagen) expression vector, for protein purification.

### Expression and purification of protein

The MMAR_2193 protein was expressed as a C-terminal hexa-histidine tagged protein in the BL21/ (DE-3) strain of *E*.*coli*. The recombinant *E*.*coli* BL21 (DE3) cells harbouring expression plasmid was grown in Luria Bertini broth incubated at 30°C until the optical density at 600 nm reached 0.5 units. It is followed by the induction of culture using the 1mM final concentration of isopropyl-1-thio-*β*-D-thiogalactopyranoside (IPTG) and incubated at 22°C. After 16h, the culture was harvested to pellet down the cells, and the bacterial pellet was re-suspended in lysis buffer (50mM Tris, pH 8.0 with 10% glycerol, 0.15M NaCl). The recombinant protein was released by sonication (30s cycle, 10s rest, 15 cycles at 30%amplitude) and the lysate formed was centrifuged at 17000rpm for 40 min at 4˚C to remove cell debris. The supernatant was collected and used for binding with 0.5ml Ni-NTA slurry. Unbound protein was washed with wash buffer (50mM Tris pH 8.0, 10% glycerol). The Ni-NTA bound protein was eluted through the imidazole gradient using gravity flow from 5mM to 150mM. SDS-PAGE was used to check the purified protein elution (**see Fig 3D**). For the coenzyme assay, MtbPKS18 was purified using Ni-NTA based affinity purification.

### Generation of methylated products and polyketide assay

Enzyme activity assay for methyltransferase was carried out using reaction mixture consisting of three probable phenolic products (resorcinol, phloroglucinol, and olivetol) of type III PKS as a substrate based on the result of cDocker and and cDocker interaction energy. The probable substrates and products are docked on to the model of MMAR_2193 with S-adenosyl-*L*-homocysteine after the protein and ligand preparation to predict the substrate for *in vitro* work.

The *in-vitro* reaction was performed for MtbPKS18, a type III PKS together with MMAR_2193, a methyltransferase. C-chain starters: C_16_-CoA with an extender malonyl (C_2_)-CoA were used for the first reaction with MtbPKS18 to get type III polyketide products. The enzymatic reaction was carried out using 100μM each starter molecule (C16-CoA) and 100μM malonyl-CoA as extender molecule. The enzymatic reactions were carried using 50μg of purified Mtbpks18 at 30˚C for 120 min. The reaction was quenched using 5% acetic acid, and the products were extracted using 2×300μl of ethyl acetate. The extracts were dried using a vacuum and were resuspended and dissolved using 10% ethanol in 50mM HEPES buffer. The products obtained from the first reaction were used as a starter substrate for the second sequential reaction getting *O*-methylated products.

Methyltransferase assay was done using a combination of 50mM extracted products from first reaction or commercially available standard resorcinol, olivetol and phloroglucinol as starter with 400μM *S*-adenosyl *L*-methionine (SAM) as methyl donor in a reaction buffer (50mM HEPES, 10mM MgSo4.7H2O, 0.1% BSA, pH 7.4), and 0.9495 mg/ml of the protein. Control reactions were kept without enzyme. Reactions were incubated at 30°C for 8h. Products were quenched by adding 5% of acetic acid. Products were extracted with the 2×equal volume of ethyl acetate was added to the reaction mixture and dried under vacuum. The extracts from the assay were resuspended using 50μl methanol. The methylated polyketide products from standard commercially available resorcinol, olivetol and phloroglucinol was further fractionated in UFLC, and that from the sequential reaction was directly characterized using SCIEX Triple TOF 5600 high-resolution mass spectrometry (HRMS)

UFLC analysis: Reaction products were resolved in reverse phase column (ES Industries, Sonama C5, 5μ 100°A; 25cm×4.6mm). A shallow gradient of 5% CH3CN in water (each containing 1% acetic acid) to 30% CH3CN over 5 min, 60% CH3CN in 15 min, 80% CH3CN in 25 min, 90% CH3CN in 35 min, and 100% CH3CN in 45min was used for separation of the products from the assay. Methylated products were characterized using SCIEX Triple TOF 5600 HRM.

### Metabolomic analyses of mycobacterial biofilms

*M*. *marinum* wild-type biofilm pellicles were grown from 1% inoculum of primary culture in triplicate using (150 mm ×25 mm) sterile polystyrene coated cell culture dish (SIGMA) with 70 ml Sauton’s fluid medium base supplemented with glucose (2%) and glycerol (2%) and was incubated for 14 days. The pellicle from biofilm was scraped out and resuspended in 100 mM Tris, pH 8.0 and further acidified to pH 4.0. The metabolites were extracted with the 2×equal volume of ethyl acetate from the acid-based hydrolysed mixture and dried under vacuum. The extracts from the assay were resuspended using 200μl methanol. Methylated products were characterized using SCIEX Triple TOF 5600 HRMS.

### *In silico* studies

Cluster analysis of *M*. *marinum*, M complete genome (accession number: CP000854) for type III PKS cluster was done using AntiSMASH [25]. Multiple Sequence alignments of different SAM-dependent methyltransferase were done by using JalView Software Waterhouse *et al* [26] and are given in supplementary figure (with an illustration of N-terminal and C-terminal domain). The NCBI (RefSeq or GenBank) accession number of protein sequences used for sequence comparison were as follows:NodS-like (sam-dependent methyltransferase, *M*. *liflandii*), AGC62714; NodS-like (sam-dependent methyltransferase, *M*. *psudohottsii*), GAQ31737; NodS-like (SAM-dependent methyltransferase, *M*. *marinum)*, ACC40642; class I SAM-dependent (methyltransferase, *M*. *ulcerans*), WP_096371067; tansferase (*M*.*tuberculosis*), CKU58587; class I SAM-dependent (methyltransferase, *M*. *tuberculosis*), WP_070890772,; class I SAM-dependent (methyltransferase, *M*. *shinjukuense*), WP_083048499; ChainA (Echinomycin biosynthesis),4NEC_A; ChainA (Sam-dependent methyltransferase, *Metahnosarcina mazei*), 3SM3_A; Chain A (sam-dependent metyltransferase, *Pyrococcus horikoshii* Ot3), 1WZN_A.

The FASTA format of MMAR*_2193* gene sequence (ACC40642) was used as the target sequence to search for the template based on the sequence alignment with Protein Databank (PDB) database. Homology modelling of MMAR_2193 model was performed based on the crystal structure of 4NEC_A downloaded from RCSB protein databank using Biovia Discovery Studio. The model was superimposed with its template. The structure of the ligand library was generated using ChemDraw Professional 17.0. Protein and ligand were prepared for *in silico* docking. Different docked poses of protein with different ligands were generated using Biovia Discovery Studio CDOCKER tool. CHARMm-based energy forcefields were used to generate the docked score and predict the correct poses [27].

## Results

### Homology based functional analyses of MMAR_2193

**Fig 1:**
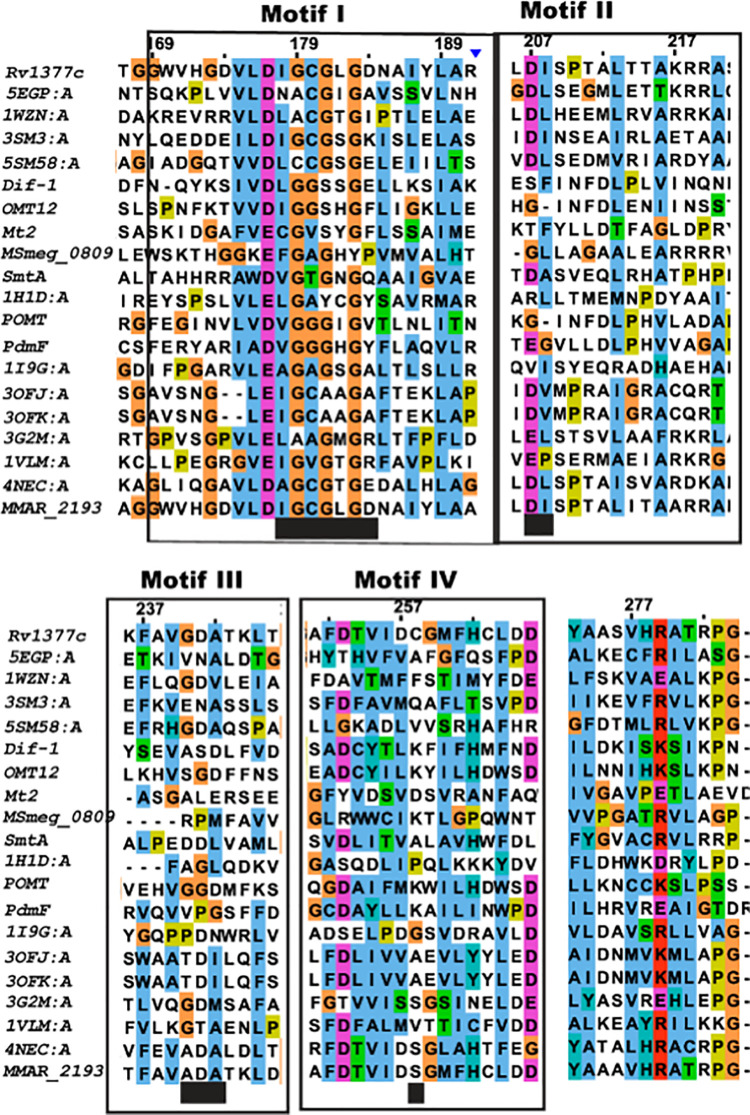
Multiple sequence alignment (MSA) of MMAR_2193. The MSA was generated to find N-terminal and C-terminal conserved amino acid for all bacterial and a few *O*-methylated representative plant O-methyltransferases (OMTs). Four conserved motifs were analyzed for MMAR_2193 generally conserved in OMTs.

Comparative sequence analysis has revealed a plethora of genomic clusters specific to pathogenic mycobacterial strains. Mmar shows a type III *pks* gene cluster with *mmar_2189* (a putative desaturase) *mmar_2190* (a putative type III *pks*), *mmar_2191* and *mmar_2192* (two probable sulfotransferase genes), followed by *mmar_2193* (a putative methyltransferase gene) as shown in **Fig 1 in S1 Text**. Our sequence-based homology comparisons predicted *mmar_2193* to belong to Nod-like SAM-dependent methyltransferase family. A comparison with RCSB protein databank entries revealed MMAR_2193 (GI: ACC40642.1) to be 37% identical (with 98% query cover) to 4NEC_A, an ADOMET methyltransferase superfamily protein that catalyzes conversion of disulphide bond into thioacetal group during echinomycin biosynthesis. Multiple sequence alignment (MSA) was carried out for MMAR_2193 with similar-length NodS–like SAM-dependent methyltransferases(AGC62714, GAQ31737), class I SAM-dependent methyltransferases (WP_096371067, WP_070890772), a transferase sequence (WP_09637106) and few structurally characterized transferases (4NEC_A, 3SM3_A, 1WZN_A). As presented in **Fig 1**, the MSA revealed conservation of functionally important domains in MMAR_2193 protein. The S-Adenosyl-*l*-methionine binding site at the N-terminal region and substrate binding site on C-terminal region of MMAR_2193 are similar to other SAM-dependent methyltransferases. The probable SAM-binding site in MMAR_2193 was identified to be conserved across the sequence in four amino acid motif positions, 49IGCGLGD55, 71DI72, 96ADA98, and 114S.

**Fig 2:**
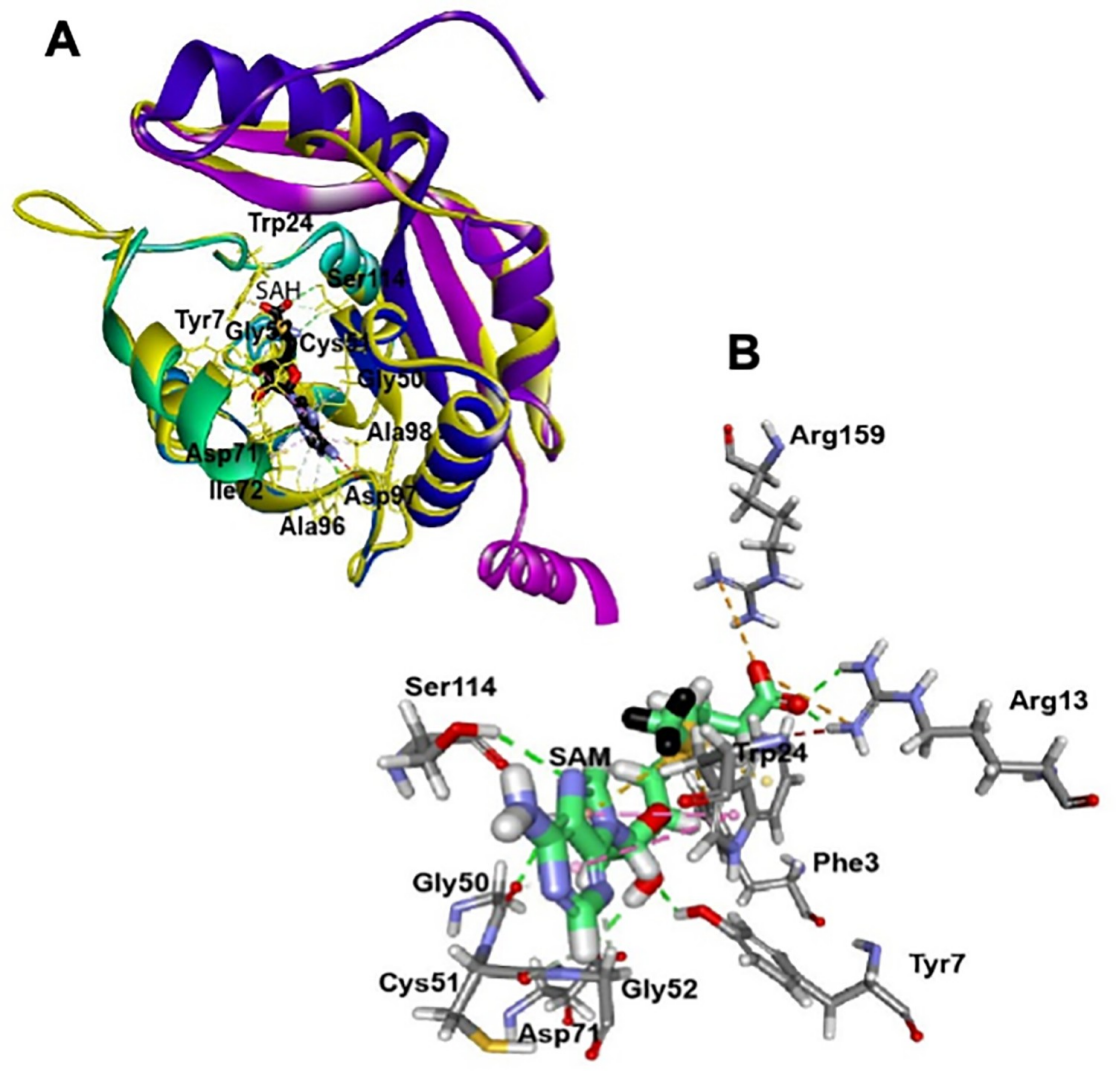
Homology model of MMAR_2193 with interacting *S*-adenosyl homocysteine (SAH) and *S*-adenosyl *L-*methionine (SAM). Template structure of 4NEC_A from Protein Data Bank (PDB) was used to generate homology model using Biovia Discovery study version 4.5. The interaction between protein and methyl donor, SAM (B) and reduced SAH (A) was derived using CHARM based force field. Methyl group of SAM is shown in black color.

In an attempt to predict the probable substrates and possible products of the protein, we carried out computational homology modeling of MMAR_2193. The model was made using ligand-bound 4NEC_A as the structural template. As depicted in **Fig 2A and 2B**, the residues interacting with SAH in 4NEC_A protein (03F07Y24W48D50GC, 71DI72, 96ADAT99, and 114S) are similar to the motifs interacting with the docked methyl donor, SAM, in MMAR_2193 model.

**Fig 3:**
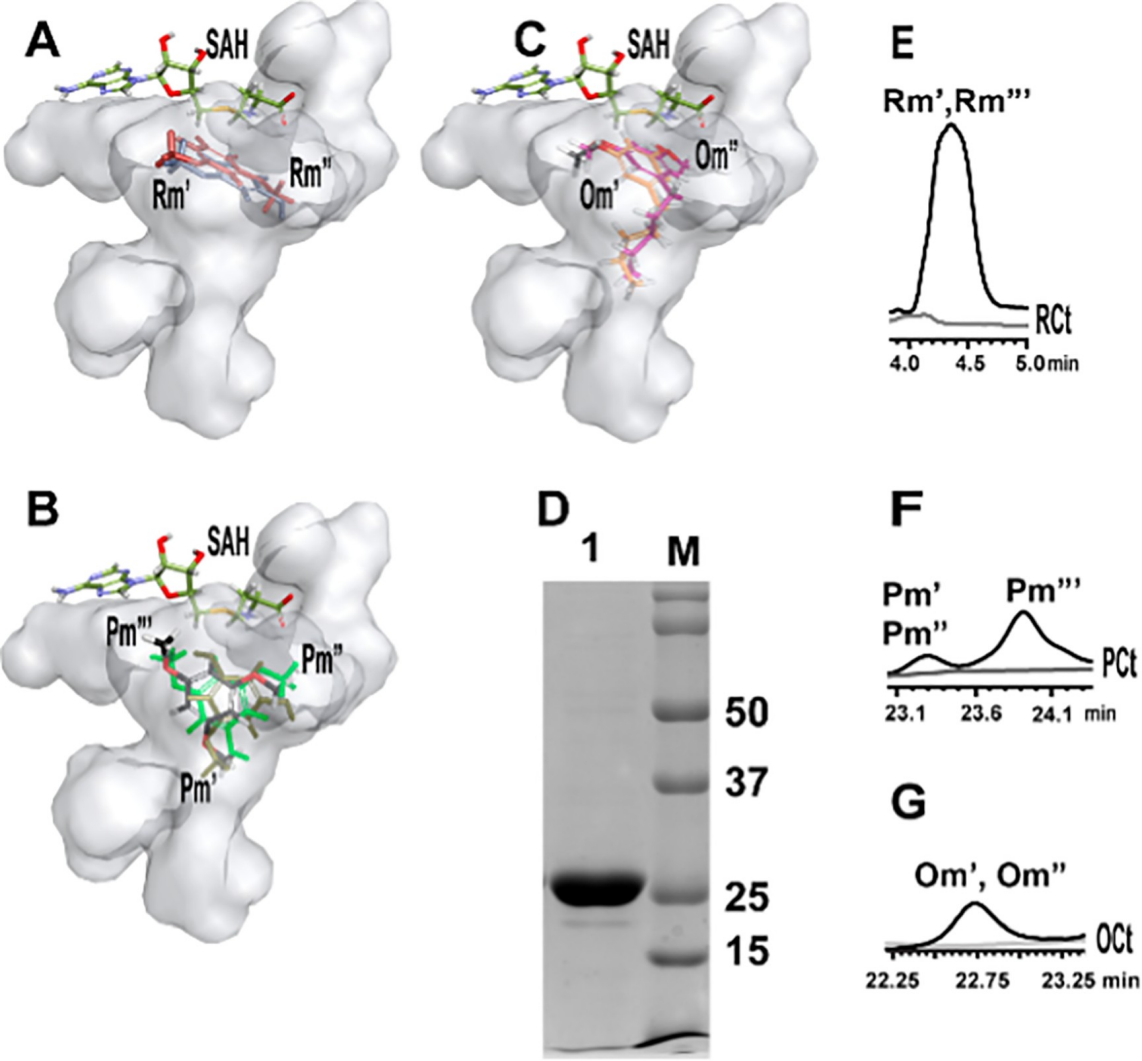
Cavity volume comparison of ligand/ products binding cavities of MMAR_2193 and fractionation of methylated products using Ultra-Fast Liquid Chromatograph (UFLC). Homology model of MMAR_2193 was used to study cavity volume near SAH binding cavity for fitting of methylated products Rm’ and Rm” (A) of resorcinol, methylated products Om’ and Om” (B) of olivetol, and methylated products Pm’, Pm” and Pm”‘ (C) of phloroglucinol. (D) shows the purified protein with approximately 25kDa size. (E), (F) and (G) display the fractionation of methylated polyketide products with respect to the controls RCt, PCt, and OCt. (E) shows the fractionation of mono- and di-methylated (Rm’ and Rm”) resorcinol together in 4 min. (F) shows the fractionation of mono- and di-methylated (Pm’ and Pm”) phloroglucinol from 23.1 to 23.4 min and fractionation of tri-methylated (Pm”‘) phloroglucinol at 24.1 min. (G) shows the fractionation of mono- and di-methylated (Om’ and Om”) olivetol from 22.25 to 23.25 min.

With SAM identified as the methyl donating substrate, we went ahead to identify possible acceptor substrates for the mycobacterial protein. *mmar_2193* clusters with a type III *pks* gene in the genome. Microbial type III PKSs are known to biosynthesize several polyphenolic lipids with aliphatic extensions on cores of resorcinol/ phloroglucinol/ *α*-pyrone scaffolds [28–31]. To acertain the possibility of MMAR_2193 being a polyketide modifier, we carried out molecular docking experiments using resorcinol, phloroglucinol, 5-pentyl-resorcinol (olivetol), and triketide- and tetraketide-*α*-hexanoylpyrones as the acceptor substrates. Notably, MMAR_2193 protein model could flexibly accommodate the polyketide cores as well as methyl-substituted probable products as shown in **Fig 3A–3C**. These methylated products fitted best in the cavity volume of the protein model near SAH binding site. **Table 1 in S1 Text** enlists the docking energies and residues interacting with each probable methylated product of all the acceptor substrates analyzed. Our computational studies thus provided clues to polyketide modifying capability of MMAR_2193 protein.

### Multiple *O*-methylations by MMAR_2193

Our *in silico* studies predicted MMAR_2193 protein to catalyze *O*-methylation of varied substrates. For functional characterization, *mmar_2193* gene was amplified from Mmar genomic DNA and cloned into *E*. *coli* expression vector system. MMAR_2193 was expressed as a hexa-histidine tagged protein and purified using Ni^***2+***^-nitrilotriacetic acid affinity chromatography as a single protein band of ~25 kDa as determined on SDS-PAGE (**Fig 3D**). Comparative structural modeling and docking studies with MMAR_2193 predicted SAM-dependent catalysis for methylation of different polyketide cores. Purified recombinant MMAR_2193 was used to perform *in vitro* enzymatic assays using SAM as a methyl donor and resorcinol/phloroglucinol/olivetol as the acceptor substrates. Extracted reaction products resolved using ultra-fast liquid chromatography (UFLC) and subjected to high-resolution mass spectrometry (HRMS) corroborated *in silico* predictions. The UFLC peaks in **Fig 3E–3G** revealed methylated products from resorcinol, phloroglucinol and olivetol, respectively, in our HRMS analyses. UFLC profile in **Fig 3E** revealed ions with [M-H]- at m/z 122.9031 and m/z 136.9603 from resorcinol primed reaction. Reaction with phloroglucinol led to product ions with [M-H]- at m/z 138.9629, m/z 152.9535 and m/z 166.9535 in UFLC peaks shown in **Fig 3F**. Olivetol primed reactions formed product ions with [M-H]- at m/z 191.1410 and m/z 207.0473 in profile observed in **Fig 3G**.

**Fig 4:**
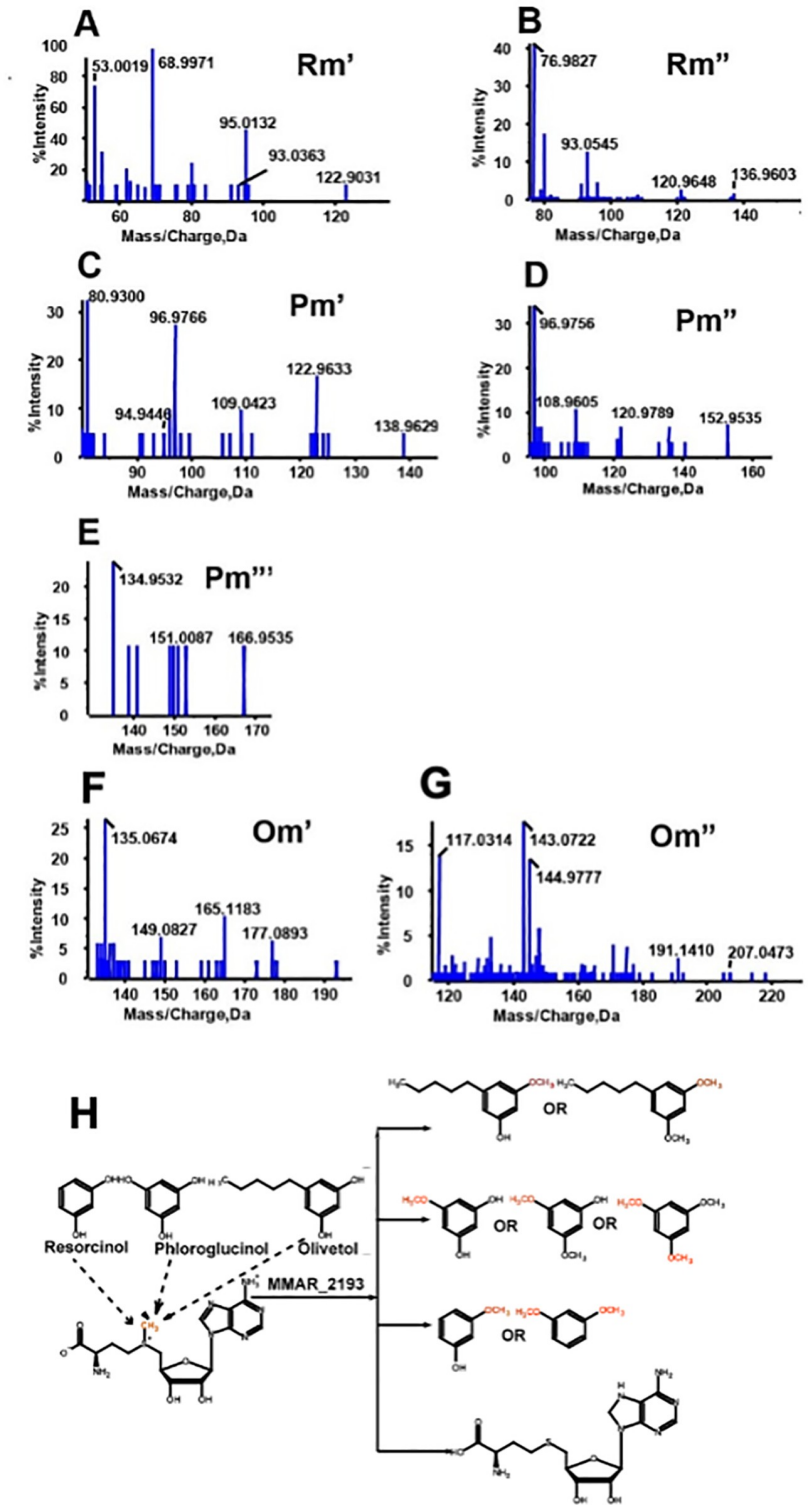
Tandem mass spectrometry for different methylated products formed from resorcinol, phloroglucinol and olivetol and overall reaction illustrating summary of methylated products formed from standard polyketide compounds. The identity of the mono- and di-methylated resorcinol (Rm’, Rm”); mono-, di- and tri-methylated phloroglucinol (Pm’, Pm” and Pm”‘) with [M-H]^-^ of m/z 122.9031 (A) and 136.9603 (B), 138.9629 (C), 152.9535 (D) and 166.9535 (E) was established by the distinctive product profile of each molecule. Tandem mass spectrometry for mono- and di-methylated olivetol (Om’ and Om”) with [M-H]^-^ of m/z 195.0995 (F) and 209.603 (G). (H) summarizes biofunctional assays of MMAR_2193 with resorcinol, phloroglucinol and olivetol using SAM as methyl donor. Methyl group is shown in red.

Tandem MS/MS analyses as shown in **Fig 4A–4G**, confirmed these product ions as mono-methylated resorcinol (Rm’, [M-H]- at m/z 122.9031: fragments at m/z 95.0132, 93.0363, 68.9971 and 53.0019); di-methylated resorcinol (Rm”, [M-H]- at m/z 136.9603: fragments at m/z 120.9648, 93.0545 and 76.9827); mono-methylated phloroglucinol (Pm’, [M-H]- at m/z 138.9629: fragments at m/z 122.9633, 109.0423, 96.9766, 94.9446 and 80.9300); di-methylated phloroglucinol (Pm”, [M-H]- at m/z 152.9535: fragments at m/z 120.9789, 108.9605 and 96.9756); tri-methylated phloroglucinol (Pm”‘, [M-H]- at m/z 166.9535: fragments at m/z 151.0087, 134.9532); mono-methylated olivetol (Om’, [M-H]- at m/z 191.1410: fragments at m/z 177.0893, 165.1183, 149.0827 and 135.0674) and di-methylated olivetol (Om”, [M-H]- at m/z 207.0473: fragments at m/z 191.1410, 144.9777, 143.0722 and 117.0314). **Table 2 in S1 Text** provides details of mass spectrometric characterization of the methylated molecules. Our functional characterization of MMAR_2193 as summarized in **Fig 4H** provided evidence for the *O*-methylation potential of the mycobacterial protein. Notably, MMAR_2193 displayed a potential to perform multiple *O*-methylations on a single substrate molecule.

### *O*-methylation of *α-*alkyl pyrones from type III polyketide synthase

**Fig 5:**
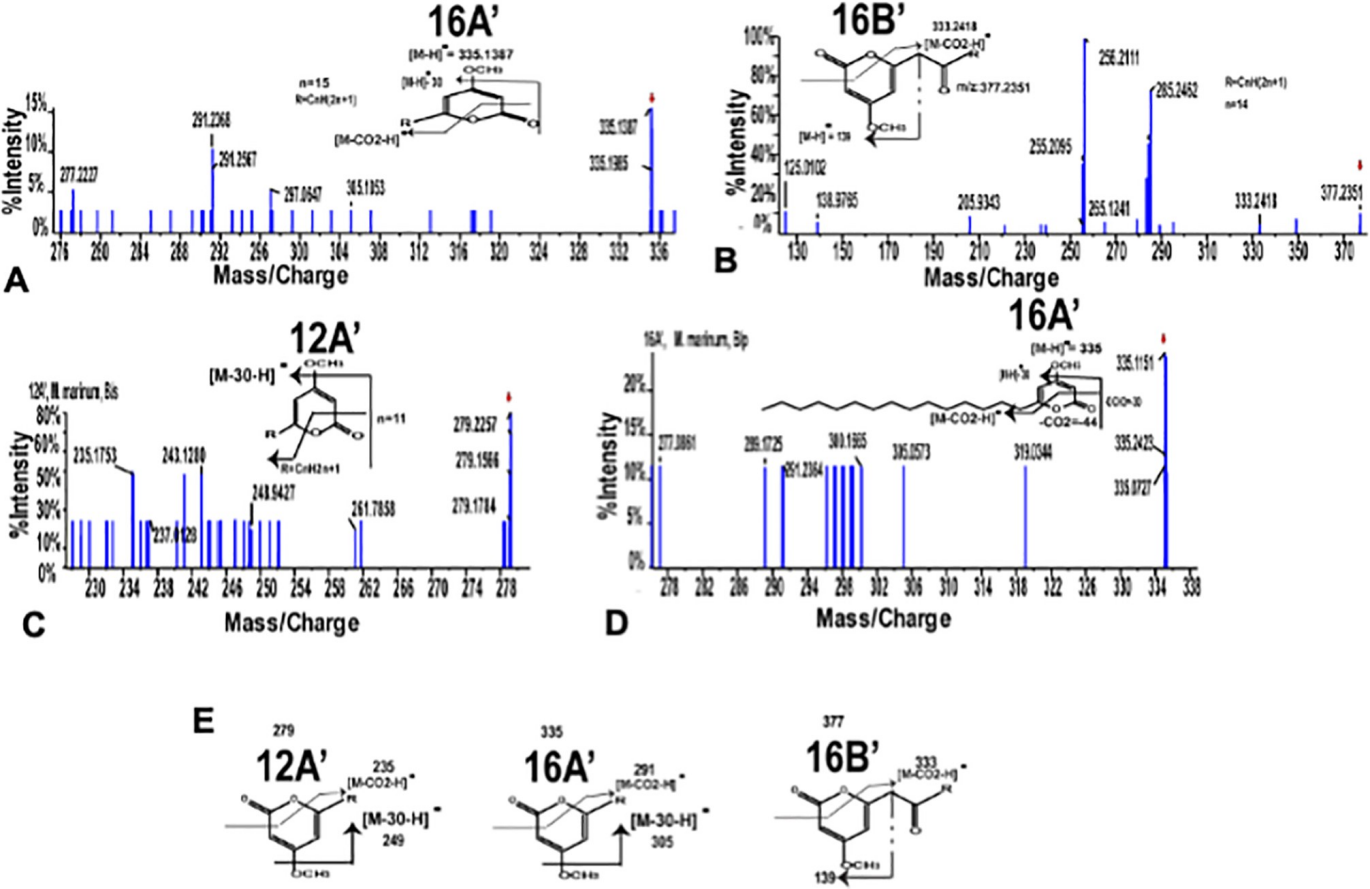
Tandem mass spectrometry of methylated *α*-triketidepyrones (16A’), *α*-tetraketidepyrone (16B’) from coupled assay and wild-type *M*. *marinum* biofilm extracts. Sequential assay using products of Mtbpks18 as substrates for methyltransferase shows formation of methylated *α*-triketidepyrone 16A’ (A) and *α*-tetraketidepyrone 16B’ (B) formed from C_16_-acyl CoA as a starter and malonyl-CoA as an extender. The metabolomics profiling of extracts from wild-type *M*. *marinum* also shows presence of *α*-triketidepyrone (12A’ and 16A’) as shown in (C) and (D). (E) shows the fragmentation pattern for the respective methylated triketide and tetraketide pyrones.

Type III polyketide synthases catalyze formation of products with diverse scaffolds. The most commonly biosynthesized products include triketide-and tetraketide-*α*-alkyl pyrones that are many times co-produced with alkyl-resorcinols or acyl-phloroglucinols. Our biochemical studies with resorcinol, phloroglucinol and olivetol provided evidence for the *O*-methylation potential of MMAR_2193. We further set out to examine MMAR_2193 mediated possible methylation of *α*-alkyl pyrones. MtbPKS18, a type III PKS from *Mycobacterium tuberculosis* has been previously characterized to catalyze biosynthesis of long-chain triketide- and tetraketide-*α-*alkyl pyrones in cell-free assays [29]. In a similar *in vitro* reaction, we incubated MtbPKS18 protein with palmitoyl-CoA (C_16_-CoA) and malonyl-CoA to biosynthesize triketide- and tetraketide-*α-*palmitoylpyrones, 16A and 16B, respectively. These metabolites were extracted from the quenched assays and used as substrates for a sequential reaction with SAM and MMAR_2193 protein. A HRMS analysis of the methyltransferase reaction products identified ions of [M-H]- at m/z 335.1987 and m/z 377.2351. Tandem MS/MS of these precursor ions confirmed the identity of these molecules as methylated *α-*alkyl pyrones, 16A’ and 16B’, respectively (**Fig 5A and 5B**). Our studies displayed the ability of MMAR_2193 to catalyze *O*-methylations on *α-*alkyl pyrone polyketides.

### *O*-methylated polyketides from mycobacterial biofilms

Mmar harbours several genomic clusters with polyketide biosynthetic genes including the MMAR_2193 cluster that is exclusively identified in pathogenic species. Biofilms have lately been associated with several pathogenic diseases [32–35] and are a natural form of existence in mycobacteria. We probed the possibility of presence of *O*-methylated polyketides in wild-type Mmar biofilms. Biofilm pellicles were developed for Mmar cells (**Fig 2 in S1 Text**) and extracted for HRMS metabolomics analyses. A multiple-reaction-monitoring (MRM) based metabolomics approach identified two ions of [M-H]^-^ at m/z 279.2257 and m/z 335.1987. Tandem MS/MS confirmed identities of *O*-methylated triketide-*α*-lauroylpyrone (12A’) and triketide-*α*-palmitoylpyrone (16A’) corresponding to the two identified ions, respectively (**Fig 5C–5E**). Our results revealed presence of *O*-methylated polyketides in wild-type Mmar biofilms.

## Discussion

Pathogenic mycobacterial genomes reveal several genomic clusters dedicated to virulent lipid biosynthesis. Polyketide synthases (PKSs) work in conjunction with fatty acid synthases to biosynthesize these molecules. Post-synthesis modification of polyketide cores is crucial for biological activity of these metabolites. Mycobacterial genomes reveal a plethora of genes homologous to methyltransferases that are important polyketide modifiers. In a distinct organization, *mmar_2193*, a probable methyltransferase was identified to be clustered with a type III *pks* and other modifying genes in pathogenic genomes. Our homology-based sequence/structure analyses predicted a SAM-dependent catalysis for MMAR_2193 protein. Structural modeling and docking studies predicted polyketide cores as probable substrates for methylation.

Interestingly, our docking studies proposed a multi-methylating potential of MMAR_2193 protein. Our biochemical studies using polyketide core molecules as substrates corroborated the *in silico* analyses. High-resolution mass spectrometry confirmed methylated polyketide products from MMAR_2193 catalyzed reactions. It was interesting to note that this mycobacterial protein utilized SAM as a donor to biosynthesize variably methylated products. Notably, MMAR_2193 exhibited the potential to methylate several hydroxyl positions on a single polyphenolic substrate molecule generating a palette of variably methylated products. Polyphenolic compounds in differing methylated states could play diverse physiological roles. MMAR_2193 utilized phloroglucinol to produce methylated products, including tri-methylated phloroglucinol or tri-methoxy benzene (TMB). TMB is the key volatile molecule that imparts typical floral scent to the Chinese rose, *Rosa chinensis*. However, the plant requires two separate classes of *O*-methyltransferases to achieve complete methylation of phloroglucinol precursor and production of TMB [36, 37]. Plant *O*-methytransferases involved in secondary metabolism generally display strict substrate specificity and through methylation direct small molecules into various metabolic pathways [15, 18, 19, 38–41]. Attempts to generate chimeric *O*-methytransferase proteins have been shown to change substrate specificity though with limited region-selectivity [42–44]. Recently, a catechol *O*-methytransferase from *Mycobacterium tuberculosis* was reported to display promiscuous substrate specificity and relaxed region-selectivity [45]. The protein however, could generate only mono-methylated products.

*O*-methyltransferases occur in several secondary metabolite generating genomic clusters. A sequencial assay of MMAR_2193 with reaction products of MtbPKS18 generated methylated *α*-pyrones *in vitro*. Based on the genomic placement of *mmar_2193* in a type III *pks* gene cluster and further the capability of the protein to methylate polyketide cores and products, it is tempting to speculate that MMAR_2193 could play crucial roles in modifying type III polyketides in *M*. *marinum*. Interestingly, *O*-methylated triketide *α*-pyrones could be identified in *M*. *marinum* biofilms, suggesting roles of these molecules in mycobacterial physiology. This study provides functional analyses of an unusual *O*-methyltransferase from *M*. *marinum*. The distinctive ability of MMAR_2193 to perform multi-methylations on varied substrates could be utilized to generate a palette of novel methylated bioactive metabolites.

## Conclusion

*O*-methyltransferase, *mmar_2193* belongs to a part of type III *pks* cluster found exclusively in pathogenic bacterial strains. Efficient *O*-methylation of hydroxyl groups seems to be essential to produce varied methylated type III PKS products. Our study reveals multiple *O*-methylating potential of MMAR_2193 to methylate all hydroxyl positions on a given substrate. *O*-methylation is reported to determine bacterial pathogenicity and survival in adverse conditions. The presence of *O*-methylated products in biofilm culture of wildtype *M*. *marinum* suggest the importance of *O*-methylation. Further, the enzyme can be utilized for generation of novel methylated scaffolds.

## Supporting information

S1 Text A supporting information file containing supporting figures and tables. (DOCX)

S1 Raw images (PDF)
