# Supplementary material for: Mycobacterial MMAR_2193 catalyzes O-methylation of diverse polyketide cores
Source: PLoS One. 2022 Jan 5;17(1):e0262241. doi: 10.1371/journal.pone.0262241 (PMC8730385; doi:10.1371/journal.pone.0262241)
Supplement: S1 Text — (DOCX) [file pone.0262241.s001.docx]

**Mycobacterial MMAR_2193 catalyzes *O*-methylation of diverse polyketide cores**

Short Title: *O*-methylation by MMAR_2193

Gorkha Raj Giri^#a^ and Priti Saxena^*^

Chemical Biology Group, Faculty of Life Sciences and Biotechnology, South Asian University, Akbar Bhawan, Chanakyapuri, New Delhi-110021, India

^#a^ Current Address: Central Department of Biotechnology, Tribhuvan University, Kirtipur, Kathmandu, Nepal

^*^Corresponding author: E-mail: [psaxena@sau.ac.in](mailto:psaxena@sau.ac.in) (PS)


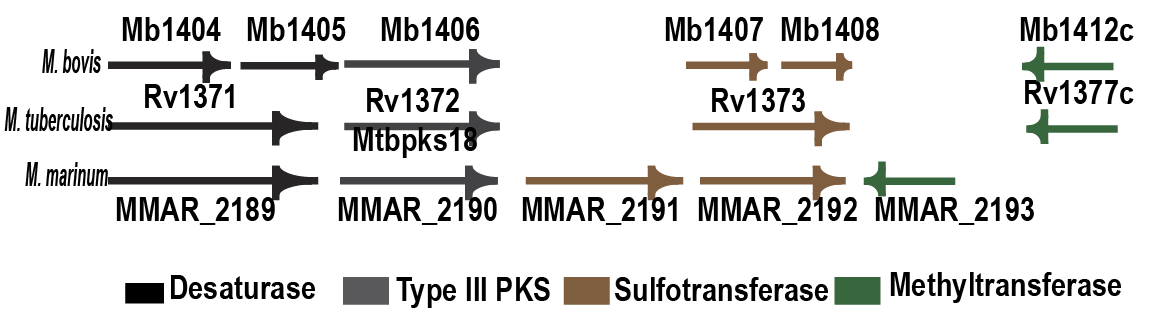


**Fig 1 in S1 text:** **Type III *pks* gene cluster**

A figure drawn to depict gene custer of *M. marinum* showing a single putative type III *pks* gene *(mmar_2190*), two probable sulfotransferases (*mma_2191*, and *mmar_2192*) and one putative methyltransferase coding gene (*mmar_2193*).


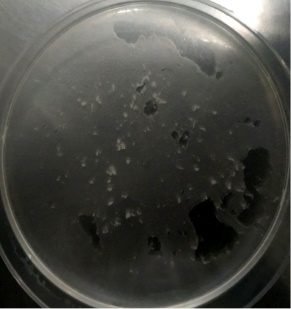


**Fig 2 in S1 text: Mmar biofilm was observed as pellicle**

Wild type species of *Mmar* is grown for pellicle study and used further for the extraction of metabolites for HRMS analyses


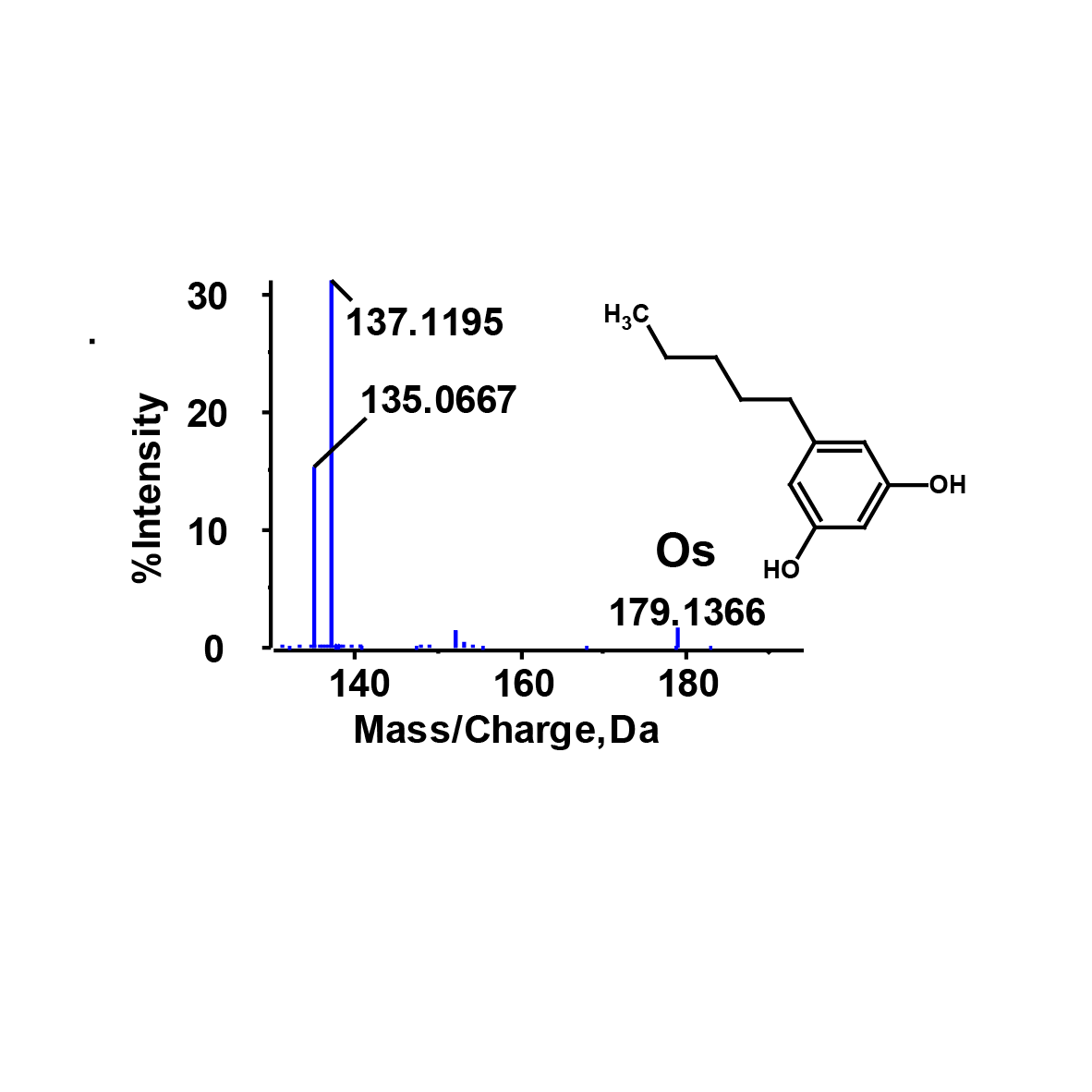


**Fig 3 in S1 text: The MS/MS fragmentation pattern of olivetol**

Similar pattern of fragmentation from tandem MS/MS mass spectrometry of olivetol (5-pentyl resorcinol) is used to understand and analyze different polyketides and modified compounds.

**Table 1 in S1 text:** Prediction of polyketide modifying capability of methyltransferase (MMAR_2193) based on the docking energies and interacting residues.


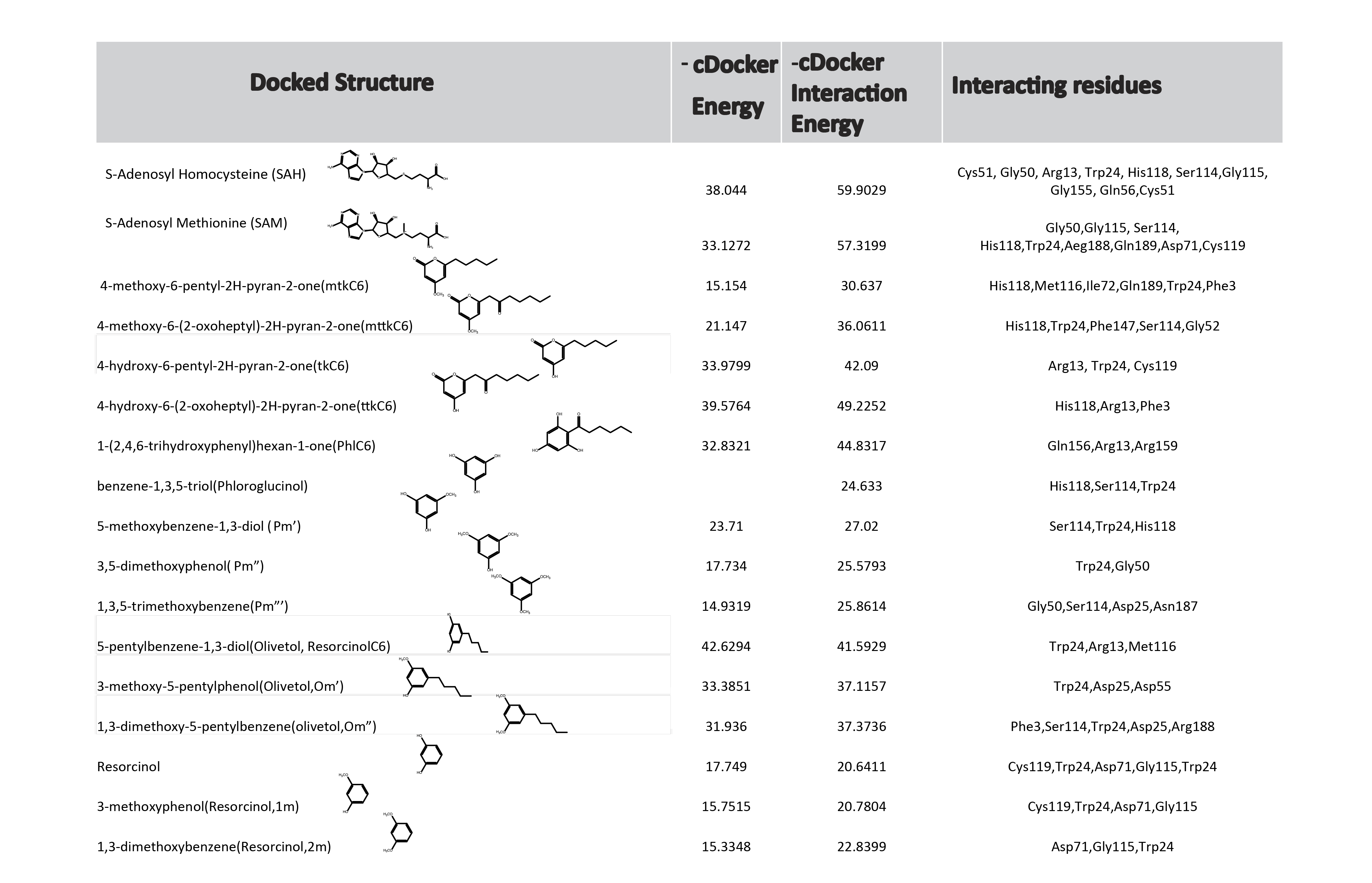


**Table 2 in S1 text:** Provides details of fragmentation pattern of all kinds of standard compounds and methylated molecules under study in the current research.

| **m/z(Ost)** | **Structure** | **Composition** | **Intensity%** | **Error Da** |
| --- | --- | --- | --- | --- |
| 135.0667  **(3)** |  | C_8_H_7_O_2_^-^ | 15.24 | 0.0222 |
| 137.1195  **(2)** |  | C_8_H_9_O_2_^-^ | 31.19 | 0.059 |
| 179.1366  (**Ost**)  **(1)** |  | C_11_H_15_O_2_^-^ | 1.57 | 0.029 |

| **m/z(Om’)** | **Structure** | **Composition** | **Intensity%** | **Error Da** |
| --- | --- | --- | --- | --- |
| 135.0674  **(6)** |  | C_8_H_7_O_2_^-^ | 26.44 | 0.022 |
| 137.0469  **(5)** |  | C_8_H_9_O_2_^-^ | 5.75 | 0.014 |
| 149.0827  **(4)** |  | C_8_H_9_O_2_^-^ | 6.90 | 0.022 |
| 165.1183  **(3)** |  | C_10_H_13_O_2_^-^ | 10.34 | 0.026 |
| 177.0893  **(2)** |  | C_11_H_13_O_2_^-^ | 6.32 | 0.003 |
| 193.1325  **(Om’)**  **(1)** |  | C_12_H1_7_O_2_^-^ | 2.87 | 0.001 |

| **m/z(Om”)** | **Structure** | **Composition** | **Intensity%** | **Error Da** |
| --- | --- | --- | --- | --- |
| 117.0314  **(5)** |  | C_8_H_5_O^-^ | 13.84 | 0.003 |
| 143.0722  **(4)** |  | C_11_H_11_^-^ | 17.68 | 0.014 |
| 144.9777  **(3)** |  | C_11_H_13_^-^ | 13.51 | 0.125 |
| 191.1410  **(2)** |  | C_13_H_19_O_2_^-^ | 2.50 | 0.033 |
| 207.0473  **(1)Om”** |  | C_11_H_19_O_2_^-^ | 0.83 | 0.092 |

| **m/z(Rst)** | **Structure** | **Composition** | **Intensity%** | **Error Da** |
| --- | --- | --- | --- | --- |
| 65.0148  **(3)** |  | C_4_HO^-^ | 100 | 0.012 |
| 67.0302  **(2)** |  | C_4_H_3_O^-^ | 9.25 | 0.011 |
| 109.0464  (**Rst**)  **(1)** |  | C_6_H_5_O_2_^-^ | 0.06 | 0.017 |

| **m/z(Rm’)** | **Structure** | **Composition** | **Intensity%** | **Error Da** |
| --- | --- | --- | --- | --- |
| 53.0019  **(5)** |  | C_3_HO^-^ | 73.33 | 0.001 |
| 55.1087  **(4)** |  | C_3_H_3_O^-^ | 31.19 | 0.000 |
| 68.9971  **(3)** |  | C_4_H_5_O^-^ | 100 | 0.038 |
| 95.0132  **(2)** |  | C_5_H_3_O_2_^-^ | 45.61 | 0.001 |
| 122.9031  **(Rm’)**  **(1)** |  | C_7_H_7_O_2_^-^ | 10.25 | 0.142 |

| **m/z(Rm”)** | **Structure** | **Composition** | **Intensity%** | **Error Da** |
| --- | --- | --- | --- | --- |
| 76.9827  **(4)** |  | C_6_H_5_^-^ | 41.41 | 0.057 |
| 93.0545  **(3)** |  | C_6_H_5_O^-^ | 12.61 | 0.020 |
| 120.9648  **(2)** |  | C_7_H_5_O_2_^-^ | 2.65 | 0.065 |
| 136.9603  **(Rm”)**  **(1)** |  | C_8_H_9_O_2_^-^ | 1.46 | 0.100 |

| **m/z(Pm’)** | **Structure** | **Composition** | **Intensity%** | **Error Da** |
| --- | --- | --- | --- | --- |
| 80.9300  **(6)** |  | C_4_HO_2_^-^ | 32.35 | 0.068 |
| 94.9446  **(5)** |  | C_5_H_3_O_2_^-^ | 4.90 | 0.069 |
| 96.9766  **(4)** |  | C_5_H_5_O_2_^-^ | 27.45 | 0.053 |
| 109.0423  **(3)** |  | C_6_H_5_O_2_^-^ | 9.80 | 0.013 |
| 122.9633  **(2)** |  | C_6_H_3_O_3_^-^ | 16.67 | 0.045 |
| 138.9629  **(Pm’)**  **(1)** |  | C_7_H_7_O_3_^-^ | 4.90 | 0.077 |

| **m/z(Pm”)** | **Structure** | **Composition** | **Intensity%** | **Error Da** |
| --- | --- | --- | --- | --- |
| 96.9756  **(5)** |  | C_5_H_5_O_2_^-^ | 34.00 | 0.054 |
| 108.9605  **(4)** |  | C_6_H_5_O_2_^-^ | 10.67 | 0.062 |
| 120.9789  **(3)** |  | C_7_H_5_O_2_^-^ | 4.00 | 0.051 |
| 136.9505  **(2)** |  | C_7_H_5_O_3_^-^ | 3.33 | 0.074 |
| 152.9535  **(Pm”)**  **(1)** |  | C_8_H_9_O_3_^-^ | 7.33 | 0.102 |

| **m/z(Pm”’)** | **Structure** | **Composition** | **Intensity%** | **Error Da** |
| --- | --- | --- | --- | --- |
| 134.532  **(3)** |  | C_8_H_7_O_2_^-^ | 23.91 | 0.092 |
| 151.0087  **(2)** |  | C_8_H_7_O_3_^-^ | 10.73 | 0.013 |
| 167.1010  **(Pm”’)**  **(1)** |  | C_9_H_12_O_3_^-^ | 10.73 | 0.013 |
