# Supplementary material for: Mycobacterial MMAR_2193 catalyzes O-methylation of diverse polyketide cores
Source: PLoS One. 2022 Jan 5;17(1):e0262241. doi: 10.1371/journal.pone.0262241 (PMC8730385; doi:10.1371/journal.pone.0262241)
Supplement: S1 Raw images — (PDF) [file pone.0262241.s002.pdf]

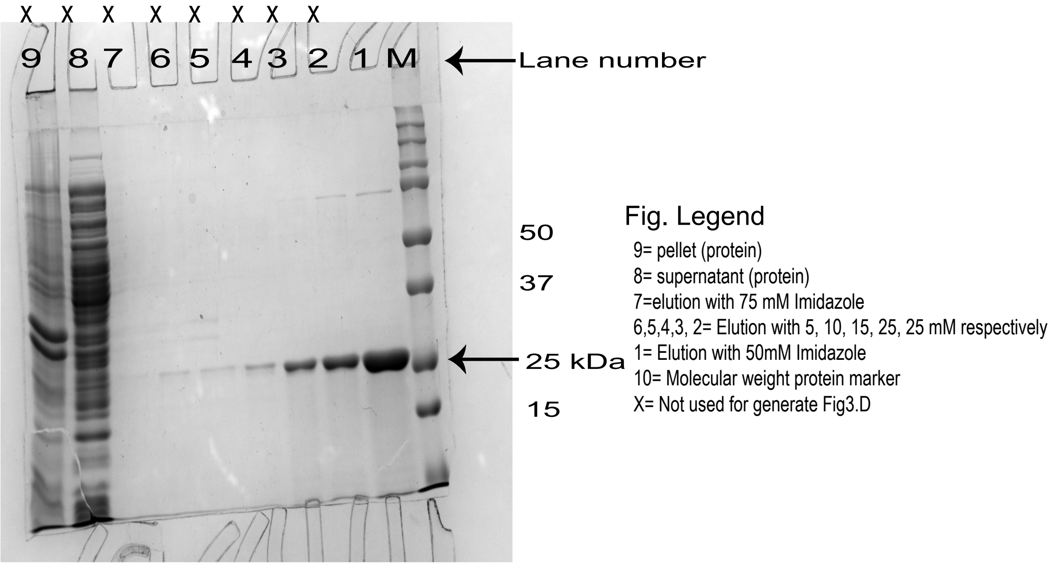

Purification of Ni-NtA purified MMAR_2193 protein using affinity chromatography

The gel picture with lane number 1 and M labelled for purified MMAR_2193 and Marker are used to generate Fig3.D
